# Supplementary material for: Quetiapine Albumin Nanoparticles as an Efficacious Platform for Brain Deposition and Potentially Improved Antipsychotic Activity
Source: Pharmaceutics. 2023 Jun 21;15(7):1785. doi: 10.3390/pharmaceutics15071785 (PMC10385742; doi:10.3390/pharmaceutics15071785)
Supplement: Supplementary file 1 [file pharmaceutics-15-01785-s001.zip › pharmaceutics-2421903-supplementary.pdf]

Supplementary data

Table S1. Model summary statistics for particle size (Y1).

Source	Std. Dev.	R^2	Adjusted R^2	Predicted R^2	PRESS	
Linear	35.51	0.6097	0.5679	0.4894	46205.03	
2FI	33.88	0.6830	0.6069	0.4606	48811.20	
**Quadratic [a]**	**13.10**	**0.9583**	**0.9412**	**0.9052**	**8580.09**	**Suggested**
Cubic	11.18	0.9793	0.9572	0.8603	12644.16	Aliased

[a] Adequate precision equals 26.73 and coefficient of variation (C.V.) % is 9.33.

Table S2. ANOVA of the obtained data from BBD for the particle size of QP-NPs and associated p-values.

Source	Sum of Squares	Df [a]	Mean Square	F-value	p-value [b]
**Model**	86715.48	9	9635.05	56.17	< 0.0001
A-HSA concentration	7196.80	1	7196.80	41.96	< 0.0001
B-pH	46016.68	1	46016.68	268.27	< 0.0001
C-Stirring time	1960.84	1	1960.84	11.43	0.0027
AB	597.42	1	597.42	3.48	0.0754
AC	13.34	1	13.34	0.0777	0.7830
BC	6015.75	1	6015.75	35.07	< 0.0001
A^2	2.80	1	2.80	0.0163	0.8994
B^2	19164.06	1	19164.06	111.72	< 0.0001
C^2	1121.53	1	1121.53	6.54	0.0180
**Residual**	3773.69	22	171.53		
Lack of Fit	3718.26	17	218.72	19.73	0.0019
Pure Error	55.43	5	11.09		
**Cor Total**	90489.18	31			

[a] Degree of freedom

[b] $^*p < 0.05$

Table S3. Model summary statistics for ziprasidone QP- NPs (Y2).

Source	Std. Dev.	R^2	Adjusted R^2	Predicted R^2	PRESS	
Linear	5.22	0.7211	0.6912	0.6289	1013.26	
2FI	5.05	0.7665	0.7105	0.5855	1131.74	
**Quadratic**	**2.26**	**0.9589**	**0.9421**	**0.9021**	**267.24**	**Suggested**
Cubic	2.05	0.9769	0.9522	0.8457	421.22	Aliased

Table S4. ANOVA of the obtained data from BBD for the EE% of QP- NPs and associated p-values.

Source	Sum of Squares	Df [a]	Mean Square	F-value	p-value [b]
**Model**	2618.22	9	290.91	57.01	< 0.0001
A-HSA concentration	1726.56	1	1726.56	338.38	< 0.0001
B-pH	198.14	1	198.14	38.83	< 0.0001
C-Stirring time	44.15	1	44.15	8.65	0.0075
AB	105.02	1	105.02	20.58	0.0002
AC	7.57	1	7.57	1.48	0.2362
BC	11.58	1	11.58	2.27	0.1461
A^2	414.43	1	414.43	81.22	< 0.0001
B^2	0.0492	1	0.0492	0.0096	0.9227
C^2	18.42	1	18.42	3.61	0.0706
**Residual**	112.25	22	5.10		
Lack of Fit	100.59	17	5.92	2.54	0.1540
Pure Error	11.66	5	2.33		
**Cor Total**	2730.48	31			

[a] Degree of freedom

[b] $*p< 0.05$

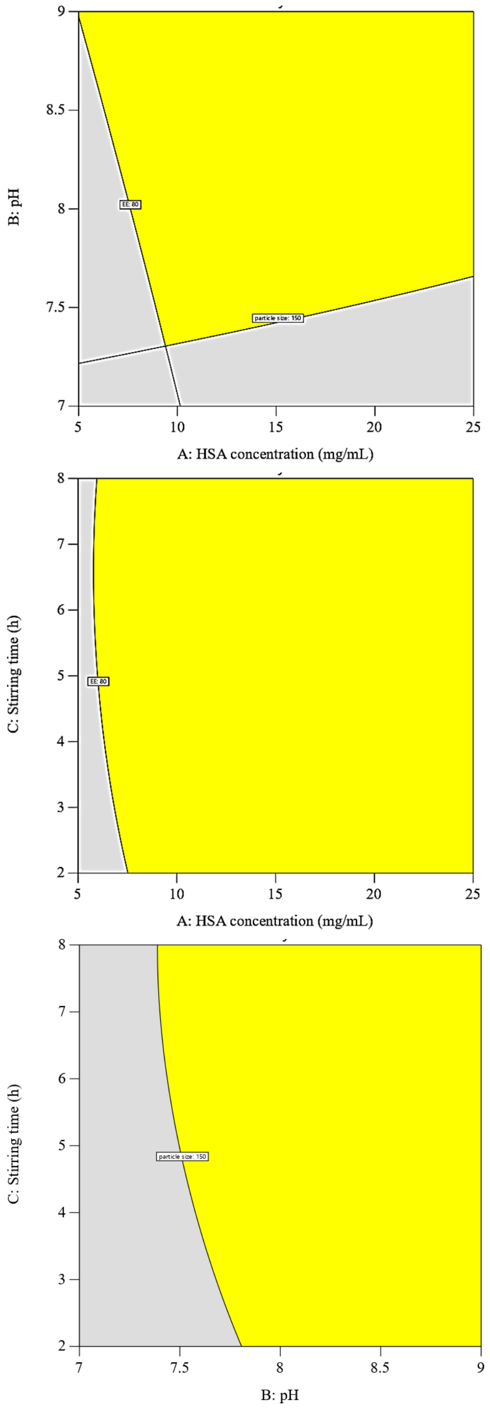

Figure S1. Overlay plots depicting the design space region for the QP- NPs. The design space was plotted by overlapping different CPPs influence on CQAs contour plots to obtain QTPP. The yellow area represents the values of CPPs when optimized to fulfill QTPP criteria; minimum particle size and maximum EE%.

Table S5. The experimental and predicted particle size and EE % of the optimized QP- NPs.

Parameter	HSA concentration (mg/mL)	pH	Stirring time (h)	Experimental	Predicted	% Pre. error	Desirability
Particle size (nm) [a,c]	18	8.6	5	103.54±2.36	97.48	5.85	0.947
EE % [b, c]				96.32±3.98	95.12	1.24	

[a] Particle size was measured by DLS.

[b] Calculated as percentage of initial quetiapine added, determined directly by HPLC.

[c] Expressed as mean ± SD (n=3).
